# Global Surgery Indicators and Pediatric Hydrocephalus: A Multicenter Cross-Country Comparative Study Building the Case for Health System Strengthening

**DOI:** 10.3389/fsurg.2021.704346

**Published:** 2021-08-26

**Authors:** Kevin Paul Ferraris, Eric Paolo M. Palabyab, Sergei Kim, Hideaki Matsumura, Maria Eufemia C. Yap, Venus Oliva Cloma-Rosales, German Letyagin, Ai Muroi, Ronnie E. Baticulon, Jose Carlos Alcazaren, Kenny Seng, Joseph Erroll Navarro

**Affiliations:** ^1^Section of Neurosurgery, Department of Surgery, Jose R. Reyes Memorial Medical Center, Manila, Philippines; ^2^Department of Pediatric Neurosurgery, Federal Neurosurgical Center of Ministry of Public Health, Novosibirsk, Russia; ^3^Department of Neurosurgery, Faculty of Medicine, University of Tsukuba, Ibaraki, Japan; ^4^ThinkWell, Manila, Philippines; ^5^101 Health Research, Makati, Philippines; ^6^Division of Neurosurgery, Department of Neurosciences, College of Medicine and Philippine General Hospital, University of the Philippines, Manila, Philippines

**Keywords:** health systems, global neurosurgery, pediatric hydrocephalus, Japan, Philippines, Russia

## Abstract

**Objective:** The aim of this study is to compare specific three-institution, cross-country data that are relevant to the Global Surgery indicators and the functioning of health systems.

**Methods:** We retrospectively reviewed the clinical and socioeconomic characteristics of pediatric patients who underwent cerebrospinal fluid (CSF) diversion surgery for hydrocephalus in three different centers: the University of Tsukuba Hospital in Ibaraki, Japan (HIC), the Jose R. Reyes Memorial Medical Center in Manila, Philippines [low-to-middle-income country (LMIC)], and the Federal Neurosurgical Center in Novosibirsk, Russia (UMIC). The outcomes of interest were the timing of CSF diversion surgery and mortality. Statistical tests included descriptive statistics, Cox proportional hazards model, and logistic regression. Nation-level data were also obtained to provide the relevant socioeconomic contexts in discussing the results.

**Results:** In total, 159 children were included, where 13 are from Japan, 99 are from the Philippines, and 47 are from the Russian Federation. The median time to surgery at the specific neurosurgical centers was 6 days in the Philippines and 1 day in both Japan and Russia. For the cohort from the Philippines, non-poor patients were more likely to receive CSF diversion surgery at an earlier time (HR = 4.74, 95% CI 2.34–9.61, *p* <0.001). In the same center, those with infantile or posthemorrhagic hydrocephalus (HR = 3.72, 95% CI 1.70–8.15, *p* = 0.001) were more likely to receive CSF diversion earlier compared to those with congenital hydrocephalus, and those with postinfectious (HR = 0.39, 95% CI 0.22–0.70, *p* = 0.002) or myelomeningocele-associated hydrocephalus (HR = 0.46, 95% CI 0.22–0.95, *p* = 0.037) were less likely to undergo surgery at an earlier time. For Russia, older patients were more likely to receive or require early CSF diversion (HR = 1.07, 95% CI 1.01–1.14, *p* = 0.035). External ventricular drain (EVD) insertion was found to be associated with mortality (cOR 14.45, 95% CI 1.28–162.97, *p* = 0.031).

**Conclusion:** In this study, Filipino children underwent late time-interval of CSF diversion surgery and had mortality differences compared to their Japanese and Russian counterparts. These disparities may reflect on the functioning of the health systems of respective countries.

## Introduction

### The Global Surgery Indicators

The Global Surgery movement has ushered in an awareness of the existing inequities in surgical care the world over (1–4). A call was made for nation-level solutions in improving access to essential surgery, in order to ultimately achieve health, welfare, and economic development by the year 2030 (1). The Global Surgery indicators, with the corresponding working definitions and targets for countries, became the standardized metrics by which the extent of the problem in the healthcare system of a country can be defined and elucidated in relation to surgical processes and outcomes (5). These six core indicators are (1) access to timely essential surgery, (2) specialist surgical workforce density, (3) surgical volume, (4) perioperative mortality, and protection against (5) impoverishing and (6) catastrophic expenditures.

### Outcome Disparities Borne From the Country of Origin and Social Determinants

Few studies have examined the association between patient-level socioeconomic factors and outcomes following treatment for pediatric hydrocephalus (6–8), and these studies are mostly situated in a single country. And yet, neurosurgical outcomes are also affected by systemic factors, particularly the availability or the absence of resources and investments within a health system. Cross-country disparities in patient outcomes are especially apparent in diseases like stroke and cancer (9, 10). The worse outcomes appear to be explained not only by patient factors but also by the perioperative care systems situated in a given hospital or country. The level of functionality of the health system of a country, which depends on the economic infrastructure (11), public policy (12, 13), responsive governance (14), and effective financing (15) arrangements, can, in turn, mitigate barriers and increase access to neurosurgical care. In this respect, the Global Surgery indicators can become useful in assessing and providing insight into the effectiveness and responsiveness of the surgical system in a neurosurgical center of a given country.

### Surgery for Pediatric Hydrocephalus as Situated in Country Contexts

Low-to-middle-income countries (LMICs) have a disproportionately larger case volume of pediatric hydrocephalus than high-income countries (HICs), owing to differences in crude birth rate and incidence of congenital and postinfectious etiologies (11, 16). The greater burden of this disease in LMICs is further compounded by the fact that access to neurosurgical care and resources for health service delivery is limited (11). As cerebrospinal fluid (CSF) diversion surgery for hydrocephalus is considered a highly equitable and cost-effective bellwether procedure (17, 18), exploring the nation-level barriers to this kind of care can provide a snapshot of the functioning of the healthcare and surgical systems of a country, and give insight into the disparities in the outcomes of patients undergoing the procedure. Using the lens of nation-level social determinants of health and the framework of Global Surgery indicators, we aim to determine whether differences in outcomes exist between specific institutions and countries of varying income levels in relation to the neurosurgical management of all-cause pediatric hydrocephalus.

## Materials and Methods

### Study Setting and Population

After approval by the institutional review boards from the three participating centers, we conducted a retrospective, cross-sectional study across countries of differing income levels. The study was conducted in three neurosurgical centers that are the hospitals of non-children: (1) the University of Tsukuba Hospital, in Tsukuba, Ibaraki, Japan, (2) Jose R. Reyes Memorial Medical Center, in Manila, Philippines, and (3) the Federal Center of Neurosurgery, Federal State Budget Institution, in Novosibirsk, Russian Federation. The population included in the study were pediatric patients admitted in a period between January 1, 2019, and December 31, 2019, at the three centers and who had either obstructive or communicating hydrocephalus with etiologies including the following but not limited to infantile-posthemorrhagic, post-infectious, congenital-structural, associated with or related to central nervous system tumors, or associated with neural tube defects like myelomeningocele. They should have undergone the minimum operation of CSF diversion, which were any one of the following: ventriculoperitoneal shunt (VPS) insertion with or without revisions, external ventricular drain (EVD) insertion, endoscopic third ventriculostomy (ETV), Ommaya reservoir insertion, or combinations thereof. Children with hydrocephalus but those who underwent another surgery without or other than CSF diversion were excluded.

### Study Variables and Other Data

Patient-level variables consisted of clinical characteristics of individual patients collected from chart review. The outcome variables were patient-level covariates: (1) time from admission-to-surgery and (2) perioperative mortality, while the explanatory variables included patient age, sex, socioeconomic status, the type of hydrocephalus, the timing of CSF diversion, and the type of CSF diversion surgery. The time to CSF diversion surgery was the outcome of interest for cross-country comparison of institutions and was subdivided into descriptive categories based on the reasonable time frames of hydrocephalus management by Mansouri and colleagues (19). Nation-level data consisted of country-specific metrics of health-system functions based on Global Surgery indicators, as well as surrogate measures of the economy and growth. Secondary data from the World Bank (20), literature on global health (21–24), and other studies of the health systems of each country (25–27) were obtained for comparison. However, these nation-level data were not subjected to statistical analyses.

### Statistical Analysis

Descriptive statistics was used to summarize the general and clinical characteristics of the participants. Frequency and proportion were used for categorical variables. The Shapiro–Wilk test was used to determine the normality distribution of continuous variables. Continuous quantitative data that did not meet the normality assumption of distribution were described using median and range. For the timing of CSF diversion surgery as a time-to-event variable, Cox proportional hazards model was used to plot the Kaplan–Meier curves. For perioperative mortality as a dichotomous variable, logistic regression was performed. The final results from regression were presented as hazard ratios and odds ratios with their associated confidence intervals. Missing data were neither replaced nor estimated. Null hypothesis was rejected at a 0.05α-level of significance. Stata 15.0 (StataCorp, College Station, TX, USA) was used for data analysis.

## Results

### Clinical and Socioeconomic Characteristics

The medical records of 159 children, 99 (62%) from the Philippines, 47 (30%) from Russia, and 13 (8%) from Japan, were reviewed (Table 1). Their median age was 2.4 (range 0–17) years, with proportions of sexes roughly similar. Using the World Bank definition of poverty, whereby those living above US$3.10 per day are “non-poor,” those living between $1.90 and $3.10 per day are “poor,” and those living below $1.90 are “extremely poor,” we found that most of the children in the Philippines belonged to poor households (77%), whereas 72 and 100% of the Russian and Japanese patients came from non-poor families, respectively. Almost 6 in 10 hydrocephalus cases in the Philippines were either post-infectious, congenital, or tumor-related. About 7 in 10 of those in Russia were infantile/post-hemorrhagic (49%) or tumor-related (21%). Two-thirds of the cases from Japan were congenital (38%) or infantile/post-hemorrhagic (23%) hydrocephalus. VPS insertion was the most common procedure for CSF diversion in the Philippine center (92%). Likewise, in the Russian center, VPS was the surgery for the majority (64%), while varied methods of CSF diversion were conducted in the Japanese center. The revision rates were 7.7, 11.1, and 25.5% in the Japanese, Philippine, and Russian centers, respectively; however, these only reflected the revisions that were done for the study duration and also included patients who were admitted for shunt dysfunction after surgery that was performed during an earlier time period.

**Table 1:** Clinical and socioeconomic characteristics of the patients.

	**Total (*n* = 159)**	**Japan (*n* = 13)**	**Philippines (*n* = 99)**	**Russia (*n* = 47)**
	**Median (Range); Frequency (%)**			
**Patient and case characteristics**				
Age (years)	2.42 (0–17)	3 (0–16)	2 (0–17)	3 (0.08–16)
Sex				
Men	75 (47.2)	5 (38.5)	46 (46.5)	24 (51.1)
Women	84 (52.8)	8 (61.5)	53 (53.5)	23 (48.9)
Socioeconomic status^*^				
Nonpoor	58 (36.5)	13 (100)	11 (11.1)	34 (72.3)
Poor	89 (56.0)	0 (0)	76 (76.8)	13 (27.7)
Extreme poverty	12 (7.5)	0 (0)	12 (12.1)	0 (0)
Hydrocephalus type				
Congenital/diagnosed prenatally	34 (21.4)	5 (38.5)	24 (24.2)	5 (10.6)
Infantile/posthemorrhagic	38 (23.9)	3 (23.1)	12 (12.1)	23 (48.9)
Postinfectious	31 (19.5)	0 (0)	27 (27.3)	4 (8.5)
Associated with myelomeningocele	14 (8.8)	2 (15.4)	11 (11.1)	1 (2.1)
Tumor-related	35 (22.0)	3 (23.1)	22 (22.2)	10 (21.3)
Others	7 (4.4)	0 (0)	3 (3.0)	4 (8.5)
CSF diversion surgery				
ETV	22 (13.8)	5 (38.5)	2 (2.0)	15 (31.9)
VPS	99 (62.3)	1 (7.7)	80 (80.8)	18 (38.3)
VPS revision	24 (15.1)	1 (7.7)	11 (11.1)	12 (25.5)
EVD	6 (3.8)	0 (0)	6 (6.1)	0 (0)
EVD + subsequent conversion to VPS	3 (1.9)	1 (7.7)	0 (0)	2 (4.3)
Ommaya	1 (0.6)	1 (7.7)	0 (0)	0 (0)
Ommaya + conversion to VPS later on	4 (2.5)	4 (30.8)	0 (0)	0 (0)
Revision surgery rate	24 (15.1)	1 (7.7)	11 (11.1)	12 (25.5)
Time from admission to CSF diversion (days)				
<24 hours (<1 day)	22 (13.8)	2 (15.4)	8 (8.1)	12 (25.5)
24–48 hours (1–2 day)	63 (39.6)	7 (53.8)	28 (28.3)	28 (59.6)
3–7	26 (16.3)	0 (0)	20 (20.2)	6 (12.8)
8–10	4 (2.5)	1 (7.7)	3 (3.0)	0 (0)
11–14	3 (1.9)	0 (0)	3 (3.0)	0 (0)
>14	41 (25.8)	3 (23.1)	37 (37.4)	1 (2.1)

* *World Bank definition of poverty: those living above US$3.10 per day are “non-poor,” those living between $1.90 and $3.10 per day are “poor,” and those living below $1.90 are “extremely poor.”*

### Primary Outcomes: Timing of CSF Diversion Surgery and Mortality

**Figure 1:**
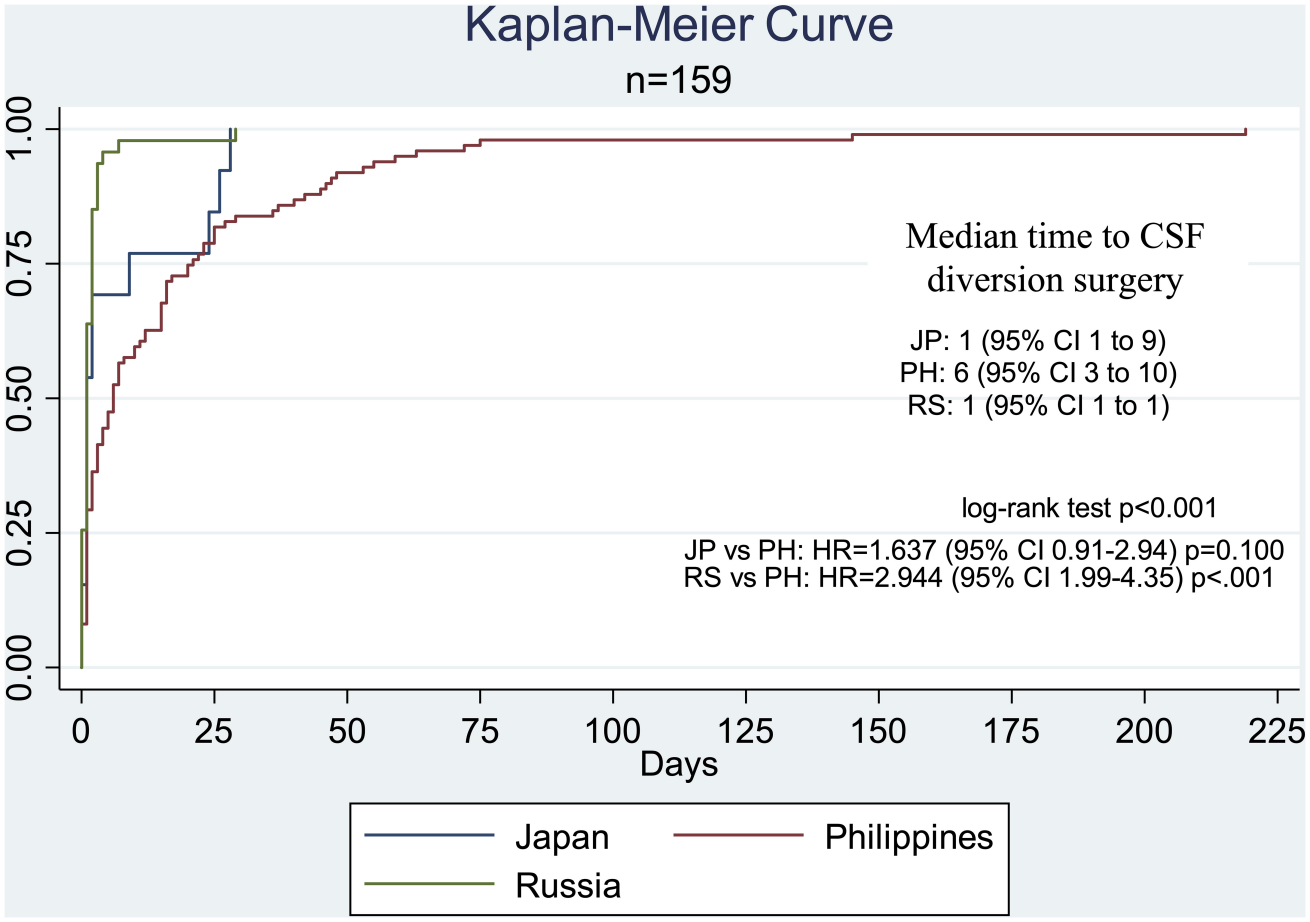
Kaplan–Meier curve comparing the time-to-CSF diversion surgery by country. The median time for the Philippine center was significantly different from that in the Russian center (HR = 2.944, 95% CI 1.99–4.35, *p* < 0.001) but not the Japanese center (HR = 1.637, 95% CI 0.91–2.94, *p* = 0.100).

For the timeliness of access to care, a majority of the cases from the Russian (85%) and Japanese (70%) centers were able to access CSF diversion within 2 days of confinement. In the Philippine center, only 36% of patients were able to undergo surgery by the second hospital day. The median time to CSF diversion was 6 days in the Philippine center, whereas it was 1 day for both the Japanese and Russian centers (Table 2; Figure 1). The delivery of surgical care for pediatric hydrocephalus was significantly more efficient in the Russian center compared to that in the Philippine center (HR = 2.94, 95% CI 1.99–4.35, *p* < 0.001). In terms of all-cause mortality, three children died in the Philippine cohort (proportion of 3.03, 95% CI 0.63–8.60), but none in the Russian and Japanese cohorts (Table 2).

**Table 2:** Primary outcome variables across different centers from the three countries.

	**Median time from admission to CSF diversion surgery**		**Perioperative mortality**	
	**Days**	**(95% CI)**	**Count (*n* = 3)**	**Proportion (95% CI)**
Japan	1	(1–9)	0	0 (0–24.7)
Philippines	6	(3–10)	3	3.03 (0.6–8.6)
Russia	1	(1–1)	0	0 (0–7.5)

### Patient-Level Determinants of Primary Outcomes

For the cohort from the Philippines, non-poor patients were more likely to receive CSF diversion at an earlier time (HR = 4.74, 95% CI 2.34–9.61, *p* < 0.001). In the same center, those with infantile or post-hemorrhagic hydrocephalus (HR = 3.72, 95% CI 1.70–8.15, *p* = 0.001) were more likely to receive CSF diversion earlier compared to those with congenital hydrocephalus, and those with post-infectious (HR = 0.39, 95% CI 0.22–0.70, *p* = 0.002) or myelomeningocele-associated hydrocephalus (HR = 0.46, 95% CI 0.22–0.95, *p* = 0.037) were less likely to undergo surgery at an earlier time. For Russia, older patients were more likely to receive or require early CSF diversion (HR = 1.07, 95% CI 1.01–1.14, *p* = 0.035). For Japan, evidence was insufficient to identify significant factors associated with the timing of CSF diversion (Table 3). Because the cohorts of Japanese and Russian children had zero mortality, only the cohort of Filipino children was analyzed for logistic regression (Table 4). EVD insertion was found to be significantly associated with mortality (cOR 14.45, 95% CI 1.28–162.97, *p* = 0.031).

**Table 3:** Determinants of the timing of CSF diversion surgery, by country, after univariate Cox regression.

	**Japan**		**Philippines**		**Russia**	
	**Crude Hazard Ratio (95% CI)**	***p-value***	**Crude Hazard Ratio (95% CI)**	***p-value***	**Crude Hazard Ratio (95% CI)**	***p-alue***
Age	1.05 (0.95–1.16)	0.334	1.00 (0.96–1.05)	0.869	**1.07 (1.005–1.14)**	**0.035**
Male sex	1.56 (0.47–5.13)	0.464	1.14 (0.76–1.69)	0.530	0.90 (0.50–1.60)	0.714
Socioeconomic status						
Poor/extreme poverty	Reference	–	Reference	–	Reference	–
Non-poor	–	–	**4.738 (2.34–9.61)**	** <0.001**	1.00 (0.52–1.91)	0.996
Hydrocephalus type						
Congenital/diagnosed prenatally	Reference	–	Reference	–	Reference	–
Infantile/posthemorrhagic	0.74 (0.16–3.41)	0.704	**3.72 (1.70–8.15)**	**0.001**	0.97 (0.36–2.62)	0.953
Postinfectious	–	–	**0.39 (0.22–0.70)**	**0.002**	1.23 (0.33–4.58)	0.761
Associated with myelomeningocele	1.26 (0.22–7.28)	0.794	**0.46 (0.22–0.95)**	**0.037**	1.52 (0.1–13.36)	0.704
Tumor-related	2.08 (0.43–9.98)	0.359	0.56 (0.31–1.01)	0.054	1.79 (0.59–5.48)	0.305
Others	–	–	2.17 (0.63–7.46)	0.217	1.47 (0.39–5.54)	0.568
CSF diversion surgery						
ETV	8.64 (0.98–76.14)	0.052	0.64 (0.16–2.63)	0.540	1.22 (0.60–2.48)	0.577
VPS	8.71 (0.49–156.26)	0.142	Reference	–	Reference	–
VPS + subsequent revision	4.78 (0.28–81.27)	0.279	0.74 (0.38–1.43)	0.367	1.17 (0.56–2.44)	0.681
EVD	–	–	1.04 (0.45–2.39)	0.934	–	–
EVD + subsequent conversion to VPS	8.71 (0.49–156.26)	0.142	–	–	0.19 (0.02–1.46)	0.110
Ommaya	4.78 (0.49–156.26)	0.279	–	–	–	–
Ommaya + conversion to VPS later on	Reference	–	–	–	–	–
Revision surgery	1.07 (0.13–8.67)	0.949	0.74 (0.38–1.44)	0.381	1.21 (0.62–2.35)	0.575

*The bold values indicate the statistical significant difference*.

**Table 4:** Determinants of mortality, in the subgroup from the Philippines, after binary logistic regression.

	**Crude Odds Ratio (95% CI)**	***pValue***
Age	0.98 (0.78–1.23)	0.872
Female sex	1.47 (0.19–11.59)	0.713
Socioeconomic status		
Poverty	4.46 (0.54–37.01)	0.166
Non-poor	Reference	–
Hydrocephalus type		
Congenital/diagnosed prenatally	Reference	–
Infantile/posthemorrhagic	1.96 (0.04–104.76)	0.740
Postinfectious	2.77 (0.11–71.35)	0.538
Associated with myelomeningocele	7.00 (0.26–186.26)	0.245
Tumor-related	3.42 (0.13–88.40)	0.459
Others	7.00 (0.12–412.69)	0.350
CSF diversion surgery		
ETV	10.60 (0.34–330.35)	0.179
VPS insertion	Reference	–
VPS insertion + revision later on	7.57 (0.72–79.62)	0.092
EVD insertion	**14.45 (1.28–162.97)**	**0.031**
Revision surgery	4.94 (0.59–41.31)	0.140
Time from admission to CSF diversion (days)	1.01 (0.99–1.03)	0.260
Time from admission to CSF diversion >14 days	12.68 (0.64–252.73)	0.096

*The bold values indicate the statistical significant difference*.

### Other Global Surgery Indicators and Social Determinants of Health

In terms of metrics that represent health-system indicants, the various indices appear to depend on and follow the trend of the income level of each respective country. The Philippines as an LMIC, when compared with the higher-income countries (Table 5), has lesser absolute numbers, proportions, and percentages of the variables that are generally accepted as social determinants of health. In terms of the Global Surgery indicators, Japan has the best neurosurgical workforce density and provides better financial risk protection as evidenced by the low out-of-pocket payment shares and a high universal health coverage (UHC) effective coverage index. The purely neurosurgical center in Russia has the highest neurosurgical volume for the duration of this study period (Table 6).

**Table 5:** Country-level data of the three countries in terms of relevant metrics of Global Surgery indicators and social determinants of health.

	**Japan**	**Philippines**	**Russia**
**Health financing and socioeconomics**			
Income level^*^	High income	Lower middle income	Upper middle income
GDP per capita, PPP (current international $, year 2019) (20)	43,235.7	9,302.4	29,181.4
Total health expenditure (as % of GDP) (23)	10.7%	4.4%	5.3%
Government and prepaid private spending on health (as % of total health spending) (23)	87.1%	44.5%	59.9%
Public health insurance and tax funding share of financing (as % of total health financing) (25–27)	84.0%	34.3%	39.4%
Out-of-pocket payment share of financing (as % of total expenditure on health) (25–27)	14.0%	53.7%	28.8%
UHC Effective Coverage Index (21)	96	55	69
**Infrastructure and workforce**			
Number of neurosurgeons	10,014	134	2,900
Neurosurgeon-to-population density	1:12,600	1:780,000	1:49,600
Hospital beds per 10,000 population (25–27)	132.0	10.1	96.8
Healthcare Access and Quality Index (22)	89	52	72

* *Based on the World Bank country classification (20), for the current 2020 fiscal year, low-income economies are defined as those with a GNI per capita, calculated using the World Bank Atlas method, of US$1,025 or less in 2018; lower middle-income economies are those with a GNI per capita between $1,026 and $3,995; upper middle-income economies are those with a GNI per capita between $3,996 and $12,375; high-income economies are those with a GNI per capita of $12,376 or more*.

**Table 6:** Institution-level data in terms of relevant metrics of Global Surgery indicators.

	**Japan**	**Philippines**	**Russia**
Institution	University of Tsukuba Hospital, Ibaraki	Jose R. Reyes Memorial Medical Center, Manila	Federal Neurosurgical Center, Novosibirsk
Institutional neurosurgical bed capacity	54	30	95
Pediatric neurosurgical bed capacity (as a share of the total neurosurgical bed capacity)	<5	<8	15
Neurosurgical volume (total number of operations during the year 2019)	714	710	4,236
Pediatric neurosurgical volume (total number of operations on children during the year 2019)	80	153	393
Neurosurgical staff (total number of consultants and residents in the year 2019)	24	12	32

## Discussion

### Hydrocephalus and Treatment Realities in Low-Resource Settings

At the Philippine neurosurgical center in this study, the usual pathway for a pediatric patient requiring CSF diversion surgery starts out with triaging the child to undergo either an elective or an emergency operation. Once the decision to shunt is made, for instance, the shunt kit will have to be sourced by the parents or caregiver from a non-profit philanthropic foundation because the catheter kits are not available at the state-financed public hospital. The time spent on shunt procurement contributes to the late time interval of surgery. Since the medical center caters primarily to the poorer segments of the population, the high volume of surgical cases coupled with limited bed capacity and constraints in the operating-room workforce also cause delays in surgery. Compounding this is the high prevalence of hydrocephalus in LMICs. These cases are commonly addressed by shunting as the procedure of choice, which is observed to have higher morbidity and mortality rates than in HICs (11, 28, 29). ETV as a procedure appears to be underutilized in the Philippine center compared to its Japanese and Russian counterparts, perhaps owing to the differences in hydrocephalus etiologies and corresponding indications, as well as variations in the institutional practice patterns. Although many studies with large patient series do not show significant differences in outcomes regardless of the kind of CSF diversion surgery employed (30, 31), or whether performed in developed or developing countries (32), the results in this cross-country comparative study show that mortality in the Philippine cohort appears to be associated with the procedure of EVD insertion. This finding, however, may largely be due to the more critical physical condition of patients who underwent EVD instead of other CSF diversion procedures. Patients who are in extremis resulting from late intervention or from perioperative complications always require resource-intensive care pathways that resource-challenged neurosurgical centers in LMICs often fail to provide. This so-called “capacity to rescue,” referring to the institutional capability to mitigate surgical risks, improve safety, and provide adequate postoperative intensive care, is the feature of quality and responsive surgical systems that can avert mortality and can improve chances of survival (10). Moreover, this statistic belies the financial and social risks that patients and their families who had unfavorable outcomes are exposed to. These risks include the high out-of-pocket expense for hospitalization, the indirect economic costs of job loss from caregiving, and their overall impoverishment even after the demise of the patient brought about by loss of livelihood, among others. In stark contrast, the Japanese university hospital and the Russian public neurosurgical center, through their highly functional national health insurance schemes and relatively well-funded surgical systems, are able to provide the families of their patients a high degree of financial risk protection from the economic shocks of hospitalization. Indeed, the undergirding health systems of the neurological centers of this study might be inextricably linked to better outcomes.

### Disparities in Health Systems: Workforce, Infrastructure, and Financing

Japan and Russia have surplus resources, better UHC, and higher public financing for health (Table 5), borne from deliberate policies of placing a prime value on public health (26, 27), and this becomes important given that the increased expenditure on surgery can easily allow the proportional expansion of surgical capacity (1, 10, 22, 33). Studies that analyzed large patient databases (34, 35) have examined the effect of nation-level socioeconomic factors on conditions treated by neurosurgeons. Remick and colleagues (34), after multivariate mixed-effects logistic regression, identified two nation-level variables, physician density and mean GDP growth, as significantly associated with good seizure outcomes following pediatric epilepsy surgery. Similarly, Guha and colleagues (35) identified a higher-country GDP and a greater neurosurgeon-to-population density as two nation-level variables that are independent predictors of good outcome following the treatment for aneurysmal subarachnoid hemorrhage. The Philippines, in contrast to the two countries, has a lower neurosurgeon workforce density (Table 5), thus expectedly restricting the breadth of access to care for children requiring CSF diversion surgery.

The increased likelihood of the inability to undergo an early CSF diversion surgery for the Filipino cohort of patients (Figure 1; Table 2), especially the poor and extremely poor subsets of Filipino patients, can be explained chiefly by financial barriers (1, 5, 19, 24, 33, 36). These barriers, direct and indirect costs relating to treatment, cause economic hardships to the household in which a patient belongs to. Families in which one of the members is a patient requiring neurosurgical care are at risk for financial catastrophe and impoverishment, and this is especially true in LMICs like the Philippines (1, 24, 28). Furthermore, out-of-pocket expenditures and the risks for catastrophic and impoverishing expenditures are higher in the Philippines than in Japan and Russia (Table 5). This is particularly disadvantageous given that our results show that a higher socioeconomic status is significantly associated with an earlier time interval of the CSF diversion surgery (Table 3). Financial risk protection, therefore, is important for the acceptability and accessibility of any surgical intervention (33), especially in countries where a significant proportion of the population is poverty-stricken (1, 21, 24, 37). This necessitates countrywide UHC for health insurance in any form or combination, precisely the kind of social structure that Japan excels at (21, 27), that would subsidize the treatment-related costs incurred by the families of the patients. While all patients from the three centers in this study have some form of health insurance coverage, those from the Japanese and Russian neurosurgical centers receive the broad range of inpatient and outpatient services with negligible out-of-pocket expenses after substantial subsidies by public insurance and government funding. These institutional features improve health-seeking behavior and provide consultations during the initial presentation of a disease more likely. Furthermore, Japan leads in having a high UHC effective coverage index (Table 5), in what could be considered as having an effective social safety net that offsets household expenditures against costly neurosurgical management (38). Financial risk protection is indeed important because inadequate or poor-quality health insurance coverage poses an increased likelihood of poor outcomes following CSF diversion procedures for pediatric patients (7, 39).

### Governance Structures and Social Determinants of Health in a Country

While a comprehensive review of the health systems of Japan (27), the Philippines (25), and the Russian Federation (26) is beyond the scope of this article, our results show that across the majority of nation-level metrics, the Philippines lags behind Japan and Russia in terms of both the Global Surgery indicators and the social determinants of health (Tables 5, 6). The provision of adequate standard of care is also shaped by the social determinants of health within a country. Several studies have shown that the economic robustness and level of resources of a country, i.e., being a HIC, translate into better patient-level outcomes, particularly when investments in perioperative care and surgical systems are made (10, 22, 34–36). Perennial lack of resources in public hospitals contributes to the inability to provide safe and quality surgical services, thus diminishing the capacity to rescue patients from avoidable deaths due to treatment complications. At the level of the neurosurgical centers, effective domestic resource mobilization in healthcare institutions is necessary for securing the health financing required to achieve improved patient outcomes. Investing in surgical services is therefore paramount, but this responsibility lies beyond the sphere of influence of organized neurosurgery.

### Policy Work and Resource Management Can Be the Way Forward

Policies that increase government expenditure on health appear to improve the composite metric that reflects the nation-level performance of a health system (22, 34). Advocacy for more strategic policies and investments that address social determinants of health can strengthen governance and financing arrangements (14, 15). These, in turn, help to reshape more responsive and equitable health and surgical systems, as certain strategies can be undertaken to reduce variations in the use of surgery (40). LMICs have the task of providing the full range of a responsive neurosurgical system, from as simple as the availability of shunt catheter kits to more capital-intensive measures such as comprehensive facility development, progressive hospital billing, strategic purchasing, and catastrophic case packages (41), that all in all curbs out-of-pocket payments and helps achieve UHC. A multilevel system approach (12, 40) by the involved policymakers can result in improvements in care processes of the surgical and health systems, which, in turn, result in a better quality of care and upon which hinges the hope of ultimately translating to better patient outcomes.

### Limitations of the Study and Future Directions

The study includes patients with considerable heterogeneity in terms of the etiology of hydrocephalus. Additionally, the neurosurgical centers are not entirely representative of their countries because of the inherent intra-national heterogeneity of institutions, especially between the public and private sectors. Selection bias and information bias may have been present because of the limitations of a retrospective review. Attributing certain outcomes to a policy when they are, in fact, owed to unmeasurable variables runs the risk of secular trend bias as well. Due to the limited sample size, the limited regression analysis, and the non-randomized and unmatched observational study design, confounding factors and their impact may not have been adequately lessened. Regardless, our study ventures into a cross-country comparison of outcomes and explores issues that are of larger socioeconomic context as related to the granularity of patient outcomes for a particular disease entity after neurosurgical intervention. If and when the Global Surgery indicator targets are met, the outcomes of neurosurgical patients in low-resource centers of LMICs after certain policy changes can be compared using the difference-in-difference study design (42). Finally, the authors look forward to increasing the center recruitment or prospectively gathering further primary patient-level data for the next phase or form of the present study. We recommend further studies with large sample sizes that allow the inclusion of nation-level covariates into a hierarchical mixed-effect statistical analysis (34, 35) that can, in turn, determine the magnitude of effect of those variables.

## Conclusion

In this study, we compared the Global Surgery outcomes following CSF diversion surgery for pediatric hydrocephalus among neurosurgical centers from different countries of varying income levels. We found that the cohort of Filipino children underwent late time interval of CSF diversion surgery compared to that of their Japanese and Russian counterparts. The differences in the timeliness of surgery were significantly related to the etiology of hydrocephalus, as well as to the socioeconomic status of the household that the child belongs to. In the cohort from the Philippines, from which three children suffered mortality, EVD insertion was associated with mortality. The variation in these outcomes may reflect the robustness of the health system of a country. Certain institution- and nation-level factors may explain the differences when viewed through the lenses of the Global Surgery indicators and social determinants of health.

## Data Availability Statement

The original contributions presented in the study are included in the article/supplementary files, further inquiries can be directed to the corresponding author/s.

## Ethics Statement

The studies involving human participants were reviewed and approved by University of Tsukuba Hospital Institutional Review Board, Jose R. Reyes Memorial Medical Center Institutional Review Board, Federal Neurosurgical Center Novosibirsk Local Ethics Committee. Written informed consent from the participants' legal guardian/next of kin was not required to participate in this study in accordance with the national legislation and the institutional requirements.

## Author Contributions

KF, SK, and HM: conception and design. EP, SK, and HM: acquisition of data. KF and VC-R: analysis and interpretation of data. KF, EP, and MY: drafting the article. KF: approved the final version of the manuscript on behalf of all authors. VC-R: statistical analysis. EP: administrative/technical/material support. KF, GL, AM, RB, JA, KS, and JN: study supervision. All authors critically revised and reviewed submitted version of manuscript.

## Conflict of Interest

VC-R is the Director of 101 Health Research. MY is a Senior Technical Advisor for ThinkWell. Both 101 Health Research and ThinkWell do not have any commercial or financial relationships with any pharmaceutical or medical device companies related to pediatric hydrocephalus. JN is Consultant and Member of the Board of Directors for Hydrocephalus Foundation of the Philippines, Inc., a non-stock, non-profit organization providing assistance for families of patients with hydrocephalus.

The remaining authors declare that the research was conducted in the absence of any commercial or financial relationships that could be construed as a potential conflict of interest.

## Publisher's Note

All claims expressed in this article are solely those of the authors and do not necessarily represent those of their affiliated organizations, or those of the publisher, the editors and the reviewers. Any product that may be evaluated in this article, or claim that may be made by its manufacturer, is not guaranteed or endorsed by the publisher.
